# Osteocalcin and C-terminal telopeptide of type I collagen in serum and saliva: A cross-sectional pilot study on correlations and implications for MRONJ risk stratification in women on antiresorptive medications

**DOI:** 10.4317/jced.64415

**Published:** 2026-08-28

**Authors:** Chitsanupong Puntrakul, Nakarin Kitkumthorn, Puangwan Lapthanasupkul, Constantin Landes, Chakorn Vorakulpipat

**Affiliations:** 1 Department of Oral and Maxillofacial Surgery, Faculty of Dentistry, Mahidol University, Bangkok 10400, Thailand; 2 Faculty of Dentistry, Burapha University, Chonburi, 20131, Thailand; 3 Department of Oral and Maxillofacial Pathology, Faculty of Dentistry, Mahidol University, Bangkok 10400, Thailand; 4 Department of Oral, Maxillofacial and Facial Plastic Surgery, Sana Klinikum, Starkenburgring 66, Offenbach am Main, Germany

**Keywords:** Medication-related osteonecrosis of the jaw, biomarkers, C-terminal telopeptide of type I collagen, osteocalcin, serum, saliva.

## Abstract

**Background:**

Medication-related osteonecrosis of the jaw (MRONJ) lacks reliable biomarkers for early detection in patients receiving antiresorptive therapy. The objective is to evaluate serum and salivary C-terminal telopeptide of type I collagen (CTX) and osteocalcin (OC) as tools to differentiate healthy controls, MRONJ at-risk patients, and those with MRONJ clinically present.

**Materials and Methods:**

Cross-sectional pilot study of 30 females categorized into controls (n=10), MRONJ at-risk (n=9), and MRONJ clinically present (n=11). Serum and unstimulated saliva were analyzed by ELISA. Statistical analyses included ANCOVA with age as covariate, Dunn post-hoc test with Bonferroni correction, and bootstrapped ROC curves (1,000 iterations).

**Results:**

After age adjustment, serum OC was a significant independent predictor of group status (p=0.002), as was salivary OC (p=0.045). Post-hoc analysis showed serum OC was significantly higher in the MRONJ at-risk group versus controls (Bonferroni-adjusted p=0.030) and MRONJ clinically present (adj p=0.012). Serum CTX did not reach significance after Bonferroni correction (p=0.183). Serum OC achieved the highest discriminatory performance (AUC=0.88, 95% CI: 0.70-1.00; 100% sensitivity at <4,801 pg/mL). No significant associations were found between biomarkers and medication duration or trigger type.

**Conclusions:**

Serum OC is a promising adjunctive biomarker for MRONJ risk stratification, maintaining diagnostic significance after age adjustment. Serum-salivary correlations suggest noninvasive monitoring potential, though serum remains the more reliable measurement medium. While the proposed cutoff (<4,801 pg/mL) serves as a valuable preliminary reference, it remains exploratory and warrants external validation in larger prospective cohorts prior to clinical application.

## Introduction

Osteoporosis, a prevalent metabolic bone disorder, features reduced bone mass, microstructural deterioration, and heightened age-related fracture risk, imposing substantial global burdens in mortality, morbidity, and economic cost [1,2]. The commonly used medications include anabolic medications such as teriparatide and antiresorptive medications such as bisphosphonates and denosumab [3], which are clinically relevant to the pathogenesis of medication-related osteonecrosis of the jaw (MRONJ), defined per 2022 AAOMS criteria as exposed maxillary/mandibular bone (probeable via intra- or extraoral fistula), persisting >8 weeks in patients with antiresorptive exposure (with/without immunomodulators/antiangiogenics), without prior jaw irradiation or metastatic disease [4]. 

Early MRONJ detection is critical to limit disease progression and improve patient outcomes. While imaging has been explored for early diagnosis, significant changes typically manifest in later stages [5,6]. Several bone turnover markers are proposed as potential predictive factors because their associations with bone remodeling processes are affected by antiresorptive medications; however, their reliability remains debated [7-10]. Studies of salivary CTX and OC have revealed differences between patients with periodontal disease and healthy controls, [11,12] suggesting a potential role for the local environment. Therefore, this study aimed to characterize the correlation between serum and salivary CTX and OC in female patients on antiresorptive medications, to delineate refined biomarker profiles capable of differentiating between healthy controls, MRONJ at-risk individuals, and those with clinically evident MRONJ.

## Materials and Methods

*Patients*

The study protocol was approved by the Faculty of Dentistry, Faculty of Pharmacy, Mahidol University, Institutional Review Board (IRB). The IRB is in full compliance with international guidelines for human research protection, including the Declaration of Helsinki, the Belmont Report, CIOMS Guidelines, and the International Conference on Harmonization in Good Clinical Practice (ICH-GCP). The IRB approval number for this study is MU-DT/PY-IRB 2022/007.2802. All procedures performed in this study were in accordance with the ethical standards of the IRB and with the Declaration of Helsinki and its later amendments. All patients were treated at the Department of the Oral and Maxillofacial Surgery, Faculty of Dentistry, Mahidol University, Bangkok, Thailand, from August 2022 to May 2024. All patients acknowledged the protocol and provided written informed consent to participate in the study.

The subjects included female patients aged 40 years or older with a plaque index score of 1 or less. The patients were divided into two major groups: The healthy control group and the antiresorptive group. The antiresorptive group was further subdivided into two subgroups: MRONJ at-risk and MRONJ clinically present in the criteria and staging of medication-related osteonecrosis of the jaw (MRONJ) 2022 American Association of Oral and Maxillofacial Surgeons (AAOMS) [4]. The sample size was calculated a priori using G*Power (version 3.1.9.7; Faul *et al*., 2007). Based on reported differences in salivary CTX levels between MRONJ-diagnosed patients (0.076 ± 0.018 ng/mL) and at-risk antiresorptive users (0.106 ± 0.025 ng/mL) in a previous pilot study with an identical three-group design (Wongla *et al*., 2021) [13], the estimated effect size was Cohen’s f=0.68. For a one-way analysis of variance with three groups (α=0.05, 1-β=0.80), a minimum of 34 participants was indicated. As this study additionally measured serum and salivary osteocalcin biomarkers not examined in Wongla *et al*. and given the rarity of MRONJ limiting recruitment, the sample was set at n=30, providing approximately 76% power for the primary ANCOVA comparison. This study is therefore considered a cross-sectional pilot investigation, and all ROC-derived cutoff values and diagnostic performance estimates should be considered exploratory, requiring external validation in larger, adequately powered cohorts.

Antiresorptive group: Patients currently on either oral or injection/infusion antiresorptive medication for at least 3 years.

MRONJ at-risk group: Patients meeting the criteria and staging for MRONJ according to the AAOMS 2022 guidelines and currently receiving either oral or injection/infusion antiresorptive medications, but without symptoms or clinical signs of MRONJ.

MRONJ clinically present group: Patients meeting the criteria and staging for MRONJ as defined by the AAOMS 2022 guidelines, exhibiting clinical signs of MRONJ in at least one location within the oral cavity, and currently receiving either oral or injection/infusion antiresorptive medications.

Healthy control group: This group included healthy patients who underwent routine dental checkups without a history of systemic diseases. 

Exclusion criteria included a history of jaw radiotherapy, evidence of metastatic disease to the jaw, the presence of medications (e.g., warfarin, steroids) or diseases that alter bone turnover marker levels (e.g., diabetes mellitus, liver disease, chronic kidney disease), other diseases limiting oral health care that contribute to impaired wound healing, and the use of tobacco products.

*Clinical procedures*

This study was designed as a single-center, cross-sectional analytic study. Patients underwent oral health examinations by an experienced dentist. Following medical history and oral hygiene assessment, eligible patients received study information, provided informed consent, and were instructed to refrain from eating, drinking, or tooth brushing for 1 hour before sample collection. Blood and saliva samples were collected in the morning (08:00-10:00) following an overnight fast of at least 8 hours, to minimize the potential influence of diurnal variation on bone turnover marker levels. Ten milliliters (mL) of blood were drawn and stored in a clot activator vacuum tube. The whole unstimulated saliva was collected via the drool method with a funnel and a sterile conical centrifuge tube until 5 mL was obtained. Because salivary concentrations of CTX and OC are substantially lower than serum, saliva samples were concentrated 30-fold using centrifugal ultrafiltration (3 kDa molecular weight cut-off; Amicon Ultra, Merck Millipore, Burlington, MA, USA) to bring analyte levels within the assay detection range. Serum samples were initially stored at -20°C and subsequently transferred to -80°C for long-term storage until batch analysis. Saliva samples were similarly stored at -20°C before the concentration procedure and then at -80°C. All samples underwent no more than one freeze-thaw cycle prior to analysis. 

*Blood and saliva sample preparation*

Samples were transported in a Styrofoam container, then centrifuged at 4°C for 10 minutes at 4700 RPM via a Thermo Scientific™ Multifuge X1 series centrifuge (Waltham, Massachusetts, USA). Serum was then collected and aliquoted. To process saliva, samples were separated and the serous component divided into 1.5 mL microvials and subjected to freeze-drying at -40°C and 0.133 millibar overnight (Labconco™ FreeZone 6 Plus, Kansas City, USA). The resulting saliva powder was then reconstituted in 50 µL of distilled water, achieving a 30x concentration. All samples were stored at -80°C until further analysis.

*Enzyme-linked immunosorbent assay (ELISA)*

C-terminal telopeptide of type I collagen

Serum CTX levels were quantified via Serum CrossLaps® (CTX-I) ELISA kit (IDS PLC; AC-02F1) following the manufacturer's instructions. 50 µL of samples, standards, and controls were added to precoated wells, followed by 150 µL of antibody solution. After sealing and 120-minute incubation at 18ºC (300 RPM), wells were washed (300 µL, 5x). Then, 100 µL of 3,3′,5,5′-tetramethylbenzidine (TMD) development solution was added, followed by 15-minute dark incubation at 18ºC (300 RPM). Finally, 100 µL of stopping solution was added, and the plate was shaken manually for 20 seconds.

*Osteocalcin*

Serum OC levels were quantified via Human Osteocalcin ELISA Kit (Abcam; ab270202) following the manufacturer's protocol. 50 µL of samples, standards, and controls were added to precoated wells, followed by 50 µL of antibody cocktail. After sealing and 60-minute incubation at 18ºC (400 RPM), wells were washed (350 µL, 3x). Then, 100 µL of TMD development solution was added, followed by 10-minute dark incubation at 18ºC (400 RPM). Finally, 100 µL of stopping solution was added, and the plate was shaken manually for 20 seconds.

Absorbances of serum and salivary CTX and OC were measured at 450 nm using a Synergy™ H1 hybrid reader with Gen5 software (BioTek, Vermont, USA). Standard curves were generated for CTX and OC, and sample concentrations were determined based on these curves.

*Statistical analysis*

Statistical analyses were performed using PASW Statistics 18 (SPSS Inc., Chicago, IL, USA) and GraphPad Prism 9.5 (Dotmatics, Boston, USA). Continuous variables are reported as medians with interquartile ranges (IQR), categorical variables as counts and percentages. Significance was determined using two-tailed tests (α=0.05). Normality was assessed via Shapiro-Wilk test. Group differences in CTX and OC were analyzed using ANCOVA with age as a covariate, with log transformations applied as needed to meet assumptions of normality, homogeneity of variances, and homogeneity of regression slopes. Post-hoc pairwise comparisons were performed using Dunn’s test with Bonferroni correction to control for Type I error inflation arising from multiple group comparisons. Bonferroni correction was applied separately within each biomarker (k=4 pairwise comparisons per biomarker; corrected significance threshold *p<*0.0125). Bonferroni-adjusted p-values are reported for all post-hoc pairwise comparisons. In figures, asterisks (*) indicate pairwise comparisons that remain statistically significant after Bonferroni correction (Bonferroni-adjusted *p<*0.0125). Correlations between CTX and OC in serum and saliva were assessed using Spearman’s rank correlation coefficient. Receiver operating characteristic (ROC) curves were generated to assess biomarker diagnostic performance for distinguishing MRONJ at-risk versus clinically present groups within the antiresorptive cohort. The 95% confidence interval (CI) for each AUC was estimated using bootstrapping (1,000 iterations with replacement, percentile method) to account for small-sample variability. Optimal cutoffs were determined by maximizing the Youden Index (YI=Sensitivity + Specificity - 1). Formal assay validation experiments (spike-recovery, dilution linearity, limit of detection, limit of quantification) were not performed for the salivary matrix in this study; the serum-based ELISA kits were applied to concentrated saliva samples following manufacturers’ instructions. This represents a methodological limitation, and salivary results should be interpreted with caution pending cross-matrix analytical validation. No data were missing for primary outcomes (serum and salivary CTX and OC levels), as all samples were successfully collected and analyzed.

## Results

*Patient characteristics*

All eligible participants meeting the inclusion criteria (n=30; 10 controls, 9 MRONJ at-risk, 11 MRONJ clinically present) were included in the study, with no exclusions due to non-participation. The demographic characteristics of the study population are reported (Table 1). The ages of these patients ranged from 61-95 years in the antiresorptive group and 40-75 years in the control group. The MRONJ triggers included tooth extraction in 5 patients (25%, mainly stage 1); spontaneous cases in 4 patients (20%, mainly stage 3); and dental implants in 2 patients (10%, stages 1 and 3). The most common antiresorptive agent was bisphosphonate (n=15 [75%]), followed by denosumab (n=3 [15%]); 2 patients (10%) used both. Oral bisphosphonates were most common (n=11 [55%]), linked to MRONJ at-risk stage in 7 patients (35%), stage 1 in 2 patients (10%), and stage 3 in 2 patients (10%). Intravenous bisphosphonates were connected to MRONJ at-risk stage in 1 patient (5%) and stage 3 in 2 patients (10%). Denosumab, administered by injection, was associated with stage 1 disease in 1 patient (5%) and stage 2 disease in 3 patients (15%). The combined use of bisphosphonates and denosumab, whether oral or injected, was sequentially given to 1 patient (5%) in each regimen.

*Serum and saliva CTX and OC levels*

No participant data were missing for the primary variables of interest; serum and salivary CTX and OC levels were successfully collected and analyzed for all 30 participants. Descriptive statistics for these biomarkers are presented as medians and interquartile ranges in Table 2.

To address the potential confounding effect of the age gap between study cohorts, an ANCOVA was performed with age as a covariate. The analysis revealed that age did not exert a statistically significant independent effect on biomarker levels (Table 3), suggesting that observed fluctuations are primarily driven by differences in clinical group status across the three study groups.

*Serum and Salivary Osteocalcin (OC)*

After adjusting for age, clinical group status remained a highly significant predictor of serum OC levels (F=8.084, *p=*0.002; Table 3). Serum OC levels were significantly elevated in the MRONJ at-risk group compared with both the control group (Bonferroni-adjusted *p=*0.030) and MRONJ clinically present group (Bonferroni-adjusted *p=*0.012). These differences remained statistically significant after correction, supporting the robustness of serum OC as a discriminating biomarker across disease stages. Furthermore, the age-adjusted model for salivary OC also demonstrated a significant group effect (F=3.490, *p=*0.045). As illustrated in Figure 1, the median level of salivary OC in the MRONJ clinically present group was lower than in the MRONJ at-risk group, though this difference did not reach statistical significance after Bonferroni correction (Bonferroni-adjusted *p=*0.150). The uncorrected p-value of 0.038 suggests a possible biological signal that may achieve significance in a larger cohort.

Serum and Salivary C-Terminal Telopeptide (CTX) 

Regarding serum CTX, no pairwise comparison reached statistical significance after Bonferroni correction (Control vs. MRONJ at-risk: Bonferroni-adjusted *p=*0.069; MRONJ clinically present vs. MRONJ at-risk: Bonferroni-adjusted *p=*0.094). The age-adjusted ANCOVA model independently confirmed that the clinical group effect on serum CTX did not reach statistical significance (F=1.817, *p=*0.183), consistent with the corrected pairwise results. Together, these findings indicate that observed differences in serum CTX levels across groups were not statistically robust after accounting for multiple comparisons and age. No statistically significant differences were observed in salivary CTX levels among the groups (*p=*0.756).

*Clinical Correlations*

Within the MRONJ clinically present group, biomarker levels were not significantly associated with either the trigger type or the total duration of antiresorptive exposure (*p>*0.05).

*Correlation analysis of CTX and OC across different parameters*

Table 4 shows no correlation between age and the levels of CTX and OC in this study. A moderate positive correlation between the serum and salivary CTX levels (*p<*0.05) in the total population was observed. In the individual groups, the correlation in the serum and saliva were moderately positive for CTX (*p<*0.05) and OC (*p<*0.05) in the antiresorptive group, but not in the control group as shown in Figure 2.

*Assessment of Diagnostic Performance for MRONJ*

The ability of each measurement to distinguish between the MRONJ at-risk group and the MRONJ clinically present group was evaluated (Table 5). The sensitivity was high (72.73%-100%), indicating a good ability to identify true positives. However, the specificity was lower (55.56%-77.78%). Serum OC exhibited superior discriminatory ability (AUC: 0.88, 95% CI: 0.70-1.00, bootstrap 95% CI: 0.70-1.00, *p=*0.004) which was greater than that of serum CTX (AUC=0.80, 95% CI: 0.59-1.00, bootstrap 95% CI: 0.56-0.96, *p=*0.03). Similarly, in saliva, OC (AUC: 0.77, 95% CI: 0.55-0.99, bootstrap 95% CI: 0.55-0.96, *p=*0.044) demonstrated superior performance compared with CTX (AUC: 0.61, 95% CI: 0.32-0.90, bootstrap 95% CI: 0.33-0.90, *p=*0.40). Overall, serum markers showed greater discriminative performance than salivary markers. Serum OC has the highest discriminative value, with a cutoff of <4801 picograms (pg)/mL, achieving 100% sensitivity and 77.78% specificity (Figure 3).

## Discussion

Estrogen deficiency causes postmenopausal osteoporosis, which increases osteoclast activity, suppresses the survival of osteocytes, and decreases osteoblast function, resulting in an imbalance in bone remodeling [14]. Bisphosphonates attenuate osteoclast function by inhibiting farnesyl diphosphate synthase in the mevalonate pathway. Their affinity for bone mineral facilitates uptake and targeted suppression of mature osteoclasts at resorption sites. Denosumab, a monoclonal IgG2 antibody, inhibits RANKL, impeding osteoclastogenesis and promoting apoptosis [15-17]. Modifications in bone remodeling, whether due to osteoporosis or antiresorptive medications, are associated with changes in bone turnover markers. Compared with premenopausal levels, bone turnover levels were found to increase in the first 10 years of menopause and then gradually decrease [18,19]. Antiresorptive medications play a role in modifying these markers, especially those related to bone resorption [20,21]; this role is particularly relevant since the majority of the antiresorptive group are postmenopausal.  In this study, neither analysis of covariance nor Spearman's rank correlation identified a statistically significant relationship between age and these key biomarker levels, indicating the complex interaction of various factors, beyond age alone. 

Osteocalcin is a protein composed of 49 amino acids that is synthesized by newly formed osteoblasts and is located primarily in the bone matrix; however, it can also be detected in serum at low concentrations. Serum osteocalcin is widely utilized as a biomarker for both bone formation and bone resorption. The incorporation of OC into the organic bone matrix allows it to be released during osteoclastic activity, thus serving as an important marker to assess metabolic bone diseases and evaluate therapeutic management in postmenopausal osteoporosis patients [22]. A positive but non-significant trend toward higher serum OC with advancing age was observed in the antiresorptive group (ρ=0.26, *p>*0.05), broadly consistent with age-related patterns reported in prior studies. Additionally, serum and salivary OC levels were lower in patients with MRONJ clinically present than in those in the MRONJ at-risk group. Notably, there was no significant difference in the median age (79 years) between the MRONJ at-risk group and those with MRONJ clinically present. These findings suggest a possible link between MRONJ clinical manifestations and reduced serum and salivary OC levels. In the context of discrimination, OC showed superior overall discriminatory ability compared with CTX in both serum and saliva, and serum OC exhibited a high sensitivity of 100% and 77.78% specificity in patients receiving antiresorptive medications at a cutoff of 4,801 pg/mL and lower.

C-terminal telopeptide of type I collagen (CTX) is a peptide fragment, derived from type I collagen telopeptides, and specifically generated during bone resorption via cathepsin K activity [22]. Historically, Marx *et al*. suggested that a serum CTX value below 100 pg/mL indicates an increased risk for MRONJ [8]. However, the clinical utility of this threshold remains controversial, as several studies have failed to find a consistent correlation between CTX levels and disease presence [7,10,23]. Our findings support this inconsistency; while a raw difference was observed, the age-adjusted ANCOVA model indicated that clinical group status was not a statistically significant independent predictor of serum CTX variance (*p=*0.183). This suggests that CTX levels may be more sensitive to systemic factors, including chronological age, than previously accounted for in clinical risk models.

The observed pattern of elevated serum OC in the MRONJ at-risk group and reduced levels in those with clinically present MRONJ may reflect distinct phases of bone metabolic response to long-term antiresorptive therapy. In the at-risk stage, despite suppressed osteoclastic activity, compensatory osteoblast activation may temporarily upregulate OC synthesis as the jawbone attempts to maintain remodeling homeostasis under continued mechanical and metabolic demands. This interpretation is consistent with the documented site-specific biology of alveolar bone, which undergoes substantially higher remodeling rates than the axial skeleton due to masticatory stress. In contrast, the decline in OC observed in the clinically evident MRONJ group may signal progressive osteoblast dysfunction or exhaustion of bone formation capacity, resulting in impaired healing. These findings align with the broader pathophysiological model of MRONJ, in which antiresorptive-induced accumulation of microdamage uncoupled from adequate reparative osteoblast activity ultimately leads to avascular necrosis. This interpretation remains hypothesis-generating and requires confirmation in mechanistic studies with histological correlation. Importantly, serum OC should be considered a potential adjunctive biomarker rather than a standalone screening tool. Its clinical utility would need to be contextualized within a comprehensive MRONJ risk assessment incorporating medication history, duration and route of antiresorptive therapy, oral infection status, local traumatic factors, mucosal findings, and radiographic changes.

Interestingly, while serum CTX levels appeared higher in the MRONJ clinically present group than in the MRONJ at-risk group on raw comparison, this difference did not reach statistical significance after Bonferroni correction (Bonferroni-adjusted *p=*0.094), and no difference was observed compared to controls. The trend toward an upward shift in markers once a lesion is present may reflect the localized inflammatory response and secondary infection associated with exposed bone, which can locally override the systemic resorptive suppression of the medication.

Regarding salivary CTX, no significant differences were detected among groups. This contrasts with findings in periodontitis research, such as those by Betsy *et al*. [11], where salivary CTX is often elevated. This discrepancy likely stems from the differing etiologies of bone destruction; while periodontitis is driven by an inflammatory response to microbial biofilms that increases remodeling, MRONJ pathology is characterized by suppressed remodeling and impaired healing. Consequently, while salivary CTX is a useful marker for periodontal inflammation, it appears to lack the sensitivity required for MRONJ risk assessment.

The attenuation of serum CTX significance after age adjustment in this study contrasts with earlier reports by Marx *et al*. [17] and Kwon *et al*. [23], who proposed serum CTX thresholds (150-300 pg/mL) as a guide for surgical risk stratification in bisphosphonate-treated patients. Several factors may account for this discrepancy. First, prior studies focused primarily on oral bisphosphonate users, whereas our antiresorptive group included intravenous bisphosphonates, denosumab, and combination regimens-agents that differ substantially in pharmacokinetics and degree of bone resorption suppression. Second, the significant age gap between our control and antiresorptive groups introduces residual confounding, as CTX is known to rise with age and menopausal status; statistical adjustment may not fully neutralize this effect in a small sample. Third, differences in sample timing, fasting status, and assay methodology across studies limit direct comparison. Finally, the cross-sectional staging used here differs from the more homogeneous surgical-risk contexts in which CTX thresholds were originally derived. These considerations collectively suggest that serum CTX alone has insufficient discriminatory power in a heterogeneous real-world antiresorptive cohort, and its utility should be re-evaluated in future studies stratified by medication type and stage.

The jawbones exhibit a significantly higher rate of bone remodeling compared to other skeletal sites, largely due to continuous mechanical stress from masticatory forces. Furthermore, the unique embryological origins of the craniofacial region influence the regulation of osteoblast transcription factors, resulting in distinct bone remodeling properties that differ from those of the axial or appendicular skeleton [24]. Intercellular signaling is notably reduced in bisphosphonate-treated jawbones compared with appendicular bones [25]. The deterioration of bone quality caused by bisphosphonates, along with the suppression of bone turnover rates, can lead to the accumulation of damage and microcracks, which may be critical triggering factors for MRONJ [26,27]. While CTX is widely used in metabolic bone disease, our findings suggest that OC provides a more robust and age-independent reflection of the localized bone environment in the context of MRONJ. Although we explored the potential influence of trigger type and duration of exposure within the MRONJ clinically present group, no statistically significant associations with biomarker levels were detected. With respect to diagnostic potential for screening between the MRONJ clinically present group and the MRONJ at-risk group, ROC analysis revealed that serum CTX achieved high sensitivity (100% at the optimal cutoff) for discriminating MRONJ at-risk from clinically present MRONJ; however, the specificity was modest (55.56%), and the overall AUC (0.80) was not statistically superior to that of serum OC. Given that CTX group differences did not survive Bonferroni correction in the primary analysis, its utility as a standalone screening tool remains limited compared with serum OC.

In patients taking antiresorptive medication, which reduces osteoclastic activity, serum CTX levels are classically expected to decrease markedly, unlike serum OC levels. In osteoporotic women, a deficiency of calcium and phosphorus can decrease the rate of bone mineralization, leading to the presence of free OC in the circulation [28]. Consequently, serum OC may serve as a superior biomarker for assessing bone remodeling activities in osteoporotic patients, as it is more abundant and its availability in circulation is less affected.

Salivary collection is a patient-friendly, noninvasive alternative to blood sampling. Our study revealed a positive correlation between the serum and salivary levels of CTX and OC in the antiresorptive group, with only CTX correlated across the entire population. Although salivary CTX was measurable, it was not significantly different among the groups. Salivary OC levels were low, which is consistent with previous findings that salivary OC is generally lower than other markers [11,12]. This may be related to how blood plasma is filtered into saliva, with CTX (900 Daltons) being much smaller than OC (5.8 kilodaltons), and lower-molecular-weight serum molecules tend to be more abundant in saliva [29]. Additionally, the rapid degradation of OC at relatively high temperatures may impact detection; however, our samples were stored at -80°C, and the preparation processes were performed at temperatures below 4°C. 

When comparing conventional serum measurements with saliva measurements as an alternative, the former demonstrated greater reliability and involved fewer procedural steps in the laboratory process. Although saliva collection is painless and well-tolerated, a larger volume of saliva is needed to obtain sufficient measurements, leading to a longer collection time than blood sampling. Additionally, saliva samples require more complex preparatory steps, which introduced extra layers of complexity and could hinder rapid or high-throughput analysis. Overall, while saliva presents a noninvasive sampling option, the limitations in biomarker levels, stability, and processing suggest that serum remains the more consistent and practical choice for reliable biomarker assessment in this context. 

This work should also be positioned relative to closely related prior studies. Peisker *et al*. examined serum CTX and osteocalcin, together with parathyroid hormone and bone-specific alkaline phosphatase, for MRONJ risk assessment, but did not include salivary measurements, a three-group risk-stratified design, or age-adjusted analysis [30]. Wongla *et al*.’s salivary CTX findings were similarly limited to a single biomarker in saliva alone [13]. By contrast, the present study provides the first simultaneous characterization of serum and salivary OC and CTX in an age-adjusted, three-group MRONJ cohort, extending both lines of prior work.

*Limitations*

Although this study observed correlations between salivary and serum bone turnover markers, challenges in measurement stemming from the inherent characteristics and variability of saliva samples introduce uncertainty regarding their application, necessitating further methodological refinements to enhance feasibility and reliability. Specifically, formal assay validation for salivary CTX and OC (spike-recovery, dilution linearity, limit of detection, limit of quantification) was not performed, and cross-matrix validity of the serum-based ELISA kits has not been established for concentrated saliva.

Given the inherent limitations of a small sample size, stemming from the low prevalence of MRONJ [4] and the logistical challenges of simultaneous blood and saliva collection, these preliminary findings warrant further investigation in larger cohorts. While the results offer promising insights into the potential utility of serum OC as a screening tool, definitive conclusions regarding the absolute validation of the test should be approached with caution. The incorporation of bootstrapping for AUC confidence interval estimation enhances the reliability of our ROC findings, particularly in this small-sample study (n=20 for the MRONJ at-risk vs. MRONJ clinically present ROC comparison). Traditional methods assume asymptotic normality, which may falter with limited data, whereas bootstrapping provides empirical distribution-based intervals without such assumptions. bootstrap CIs were slightly narrower than standard estimates for moderate performers (e.g., serum CTX: 0.56-0.96 vs. 0.59-1.00), underscoring its utility for precise inference in correlated diagnostic data. Future work could explore bias-corrected accelerated bootstrapping for even greater accuracy in small cohorts.

The control group consisted of relatively younger, healthy women (median age 47 years) compared with the antiresorptive group (median age 79 years). This age mismatch is an inherent design constraint, reflecting the clinical reality that MRONJ predominantly affects elderly patients on long-term antiresorptive therapy. Although ANCOVA was applied with age as a covariate to address this imbalance, statistical adjustment cannot fully eliminate confounding, particularly in a small sample. The absence of information on menopausal status, body mass index, vitamin D supplementation, smoking status, and duration of antiresorptive therapy are additional limitations that may have influenced bone turnover marker levels. The antiresorptive cohort intentionally reflected real-world clinical diversity, encompassing oral bisphosphonates, intravenous bisphosphonates, denosumab, and combination regimens; this breadth enhances ecological validity, though agent-specific subgroup analyses were not feasible at the current sample size and represent a clear direction for future work. As with any single-center pilot investigation, these findings require confirmation in independent, prospectively recruited, adequately powered cohorts before the proposed serum OC cutoff can inform clinical surgical decision-making. The absence of direct AUC comparison tests and external validation are acknowledged; both are planned priorities in the next phase of this research program. Notwithstanding these limitations, this study provides the first simultaneous characterization of serum and salivary OC and CTX in a three-group MRONJ cohort with age adjustment, and the identified cutoff value offers a quantitative reference point to anchor the design of future validation studies.

## Conclusions

*Clinical Implications*

Beyond its cross-sectional discriminatory value, the observed trajectory of serum OC across MRONJ clinical stages raises the possibility of its utility as a longitudinal monitoring signal. In patients on long-term antiresorptive therapy, serial serum OC measurement combined with structured oral clinical examination and radiographic review may support earlier identification of the transition from compensated antiresorptive exposure to subclinical bone compromise. A single threshold value is inherently limited in a dynamic clinical setting where MRONJ risk fluctuates with medication changes, intercurrent dental infection, and oral surgical interventions. When incorporated into a comprehensive pre-surgical assessment alongside medication history, oral infection status, local traumatic factors, and imaging findings, serum OC may serve as a useful adjunct to support individualized dental treatment planning and improve surveillance outcomes. Future prospective studies should evaluate whether declining OC trends over time, combined with clinical and radiographic risk factors, can improve MRONJ surveillance in patients receiving long-term antiresorptive therapy.

This cross-sectional pilot study demonstrates that serum osteocalcin (OC) is a promising adjunctive biomarker for differentiating MRONJ at-risk patients from those with clinically evident disease, maintaining independent diagnostic significance after age adjustment. Even after adjusting for the significant age gap between cohorts, serum OC remained a significant independent predictor of clinical status (*p=*0.002), characterized by high sensitivity and moderate specificity. While raw serum CTX levels varied across groups, their predictive value was attenuated when controlled for age. Significant positive correlations between serum and salivary OC suggest that saliva holds potential as a non-invasive medium for bone turnover monitoring, though serum remains the more precise measurement medium at this stage. The proposed OC cutoff (<4,801 pg/mL; sensitivity 100%, specificity 78%) is exploratory and must be externally validated in larger, prospective cohorts before it can inform clinical surgical decision-making. Given the small sample size and the absence of an independent validation cohort, this cutoff should be regarded as a preliminary, hypothesis-generating reference point rather than a validated clinical threshold, and it should not, on its own, guide decisions on preventive tooth extraction or other surgical management. Serum OC should be considered an adjunctive biomarker to be interpreted alongside medication history, oral examination, and radiographic findings not as a standalone screening criterion. Larger-scale longitudinal trials are warranted to validate these cutoff values, confirm the potential of serial OC monitoring, and establish the long-term clinical utility of both serum and salivary biomarkers in MRONJ risk stratification. 

## Figures and Tables

**Table 1:** Demographic and clinical characteristics of study participants in the antiresorptive and control groups.

		**Antiresorptive group** **(n=20)**	**Control group** **(n=10)**
Age: median (range) (year)		79 (61-95)	47 (40-75)
Underlying disease (n [%])	Hypertension	5 (25)	-
	Dyslipidemia	6 (30)	
	Cardiovascular disease	3 (15)	
Trigger factor (n [%])	Spontaneous	4 (20)	-
	Tooth extraction	5 (25)	
	Dental implant	2 (10)	
Antiresorptive agent (n [%])	Bisphosphonate	15 (75)	-
	Denosumab	3 (15)	
	Combination	2 (10)	
Route of administration (n [%])	Oral	11 (55)	-
	Injection	7 (35)	
	Combination	2 (10)	
AAOMS MRONJ stage (n [%])	At-risk	9 (45)	-
	Stage 0	0 (0)	
	Stage 1	5 (25)	
	Stage 2	2 (10)	
	Stage 3	4 (20)	

**Table 2:** Median, interquartile range (IQR) levels of CTX and OC in serum and saliva for each study group.

	**n**		**CTX**		**OC**	
			**Serum (pg/mL)**	**Saliva (pg/mL)**	**Serum (pg/mL)**	**Saliva (pg/mL)**
Control	10	Median	232.28	7.62	3,792.99	0.09
		Q3	337.01	16.08	4,451.68	1.35
		Q1	197.00	3.45	2,046.85	0.00
Antiresorptive	20	Median	151.95	13.25	4,217.45	0.19
		Q3	395.19	23.93	6,845.00	1.47
		Q1	134.83	2.90	2,745.10	0.00
MRONJ At-risk	9	Median	133.00	3.16	7,820.00	1.26
		Q3	167.00	25.99	9,290.00	2.52
		Q1	98.00	1.96	4,858.77	0.26
MRONJ Clinically present	11	Median	165.47	15.83	3,312.08	0.00
		Q3	450.00	17.03	4,159.24	0.55
		Q1	144.44	8.49	2,546.67	0.00

**Table 3:** Analysis of Covariance (ANCOVA) assessing the independent effects of clinical group status and age on bone turnover markers.

**Source**	**Serum CTX**		**Salivary CTX**		**Serum OC**		**Salivary OC**	
	**F**	*p-value*	**F**	*p-value*	**F**	*p-value*	**F**	*p-value*
Corrected Model	1.430	0.257	0.697	0.562	6.385	0.002	3.091	0.044
Group	1.817	0.183	0.283	0.756	8.084	0.002	3.490	0.045
Age	0.086	0.772	0.340	0.565	0.326	0.573	3.397	0.077

**Table 4:** Spearman's rank correlation coefficients for bone turnover markers.

**Correlation pair**	**Total population (** *p* **)**	**Antiresorptive Group (** *p* **)**	**Control Group (** *p* **)**
Age - CTX	-0.22		
Age - OC	0.26		
Age - S-CTX	0.25		
Age - S-OC	0.10		
CTX - OC	-0.35*	-0.55*	0.18
CTX - S-CTX	0.41**	0.54*	0.18
CTX - S-OC	-0.21	-0.52*	0.31
OC - S-CTX	-0.22	-0.24	-0.37
OC - S-OC	0.03	0.52*	-0.42
S-CTX - S-OC	0.12	-0.14	0.53*

*p<0.05, **p<0.001, S-CTX: Salivary CTX, S-OC: Salivary OC

**Table 5:** Diagnostic performance of serum and salivary CTX and OC for distinguishing MRONJ at-risk from clinically present MRONJ.

		**Cut off (pg/mL)**	**Sensitivity (%)**	**Specificity (%)**	**95% CI**	**95% CI Bootstrap**	**AUC**	*P*	**YI**
MRONJ at-risk vs. MRONJ clinically present	CTX	>134.2	100	55.56	0.59 - 1.00		0.80	0.030	0.56
		>136.7	90.91	55.56		0.56 - 0.96			0.46
		>138.2	90.91	66.67					0.58
	OC	<4,569	90.91	77.78	0.70 - 1.00		0.88	0.004	0.69
		<4,801	100	77.78		0.70 - 1.00			0.78
		<5,689	100	66.67					0.67
	S-CTX	>5.17	81.82	55.56	0.32 - 0.90		0.61	0.400	0.37
		>7.20	81.82	66.67		0.33 - 0.90			0.48
		>8.50	72.73	66.67					0.39
	S-OC	<0.20	72.73	77.78	0.55 - 0.99		0.77	0.044	0.51
		<0.62	72.73	66.67		0.55 - 0.96			0.40
		<1.10	81.82	66.67					0.48

OC: OC, S-CTX: Salivary CTX, S-OC: Salivary OC, 95% CI: 95% Confidence Interval, AUC: Area under the curve, YI: Youden Index

**Figure 1:**
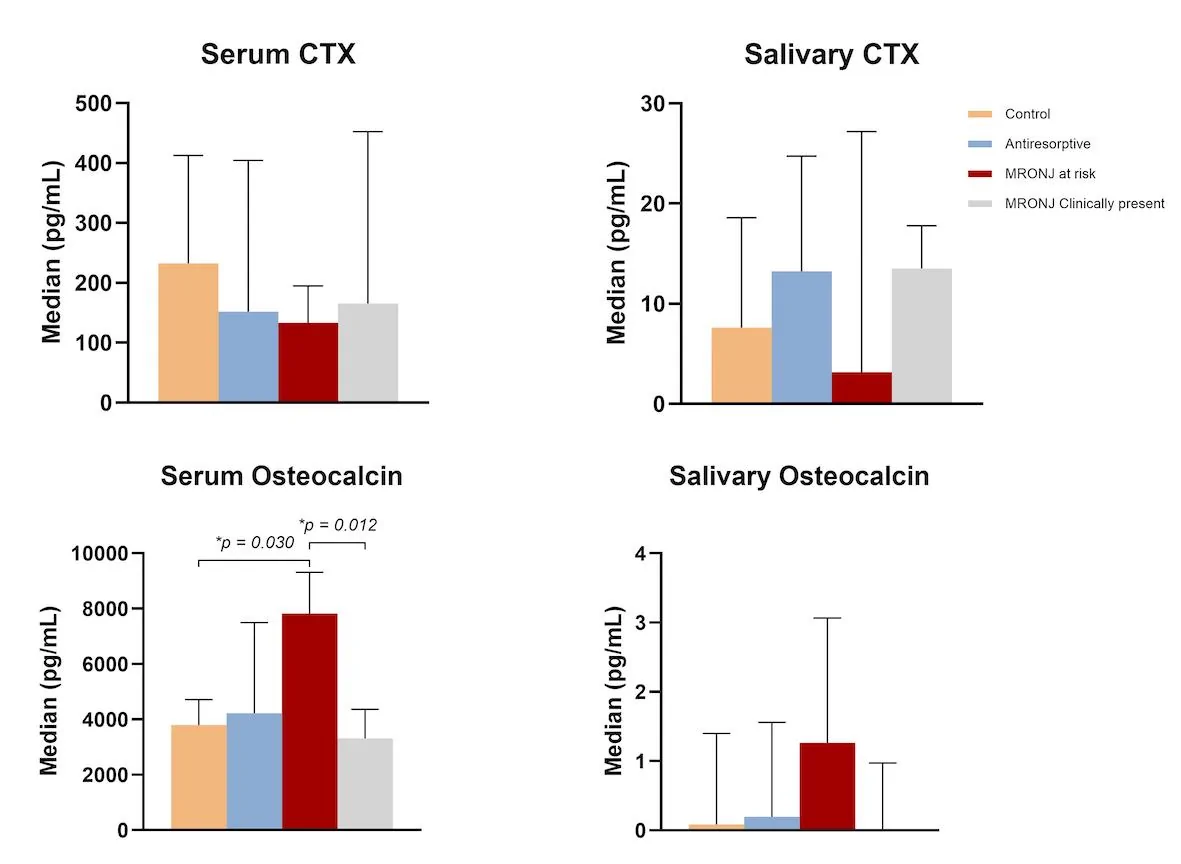
Median levels of CTX and OC in serum and saliva, comparing the control, antiresorptive, MRONJ at-risk, and MRONJ clinically present groups. P-values are indicated for significant differences between groups.

**Figure 2:**
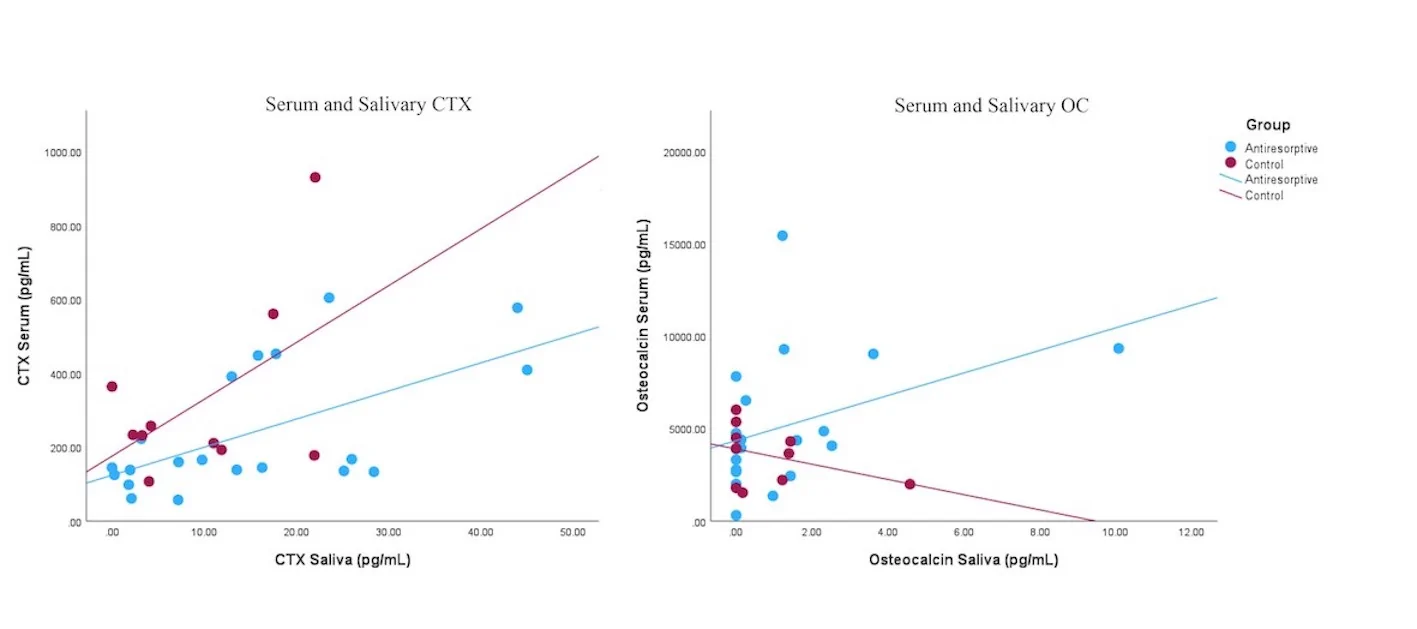
Scatter plots illustrating the correlation between serum and salivary CTX and OC levels in the antiresorptive and control groups.

**Figure 3:**
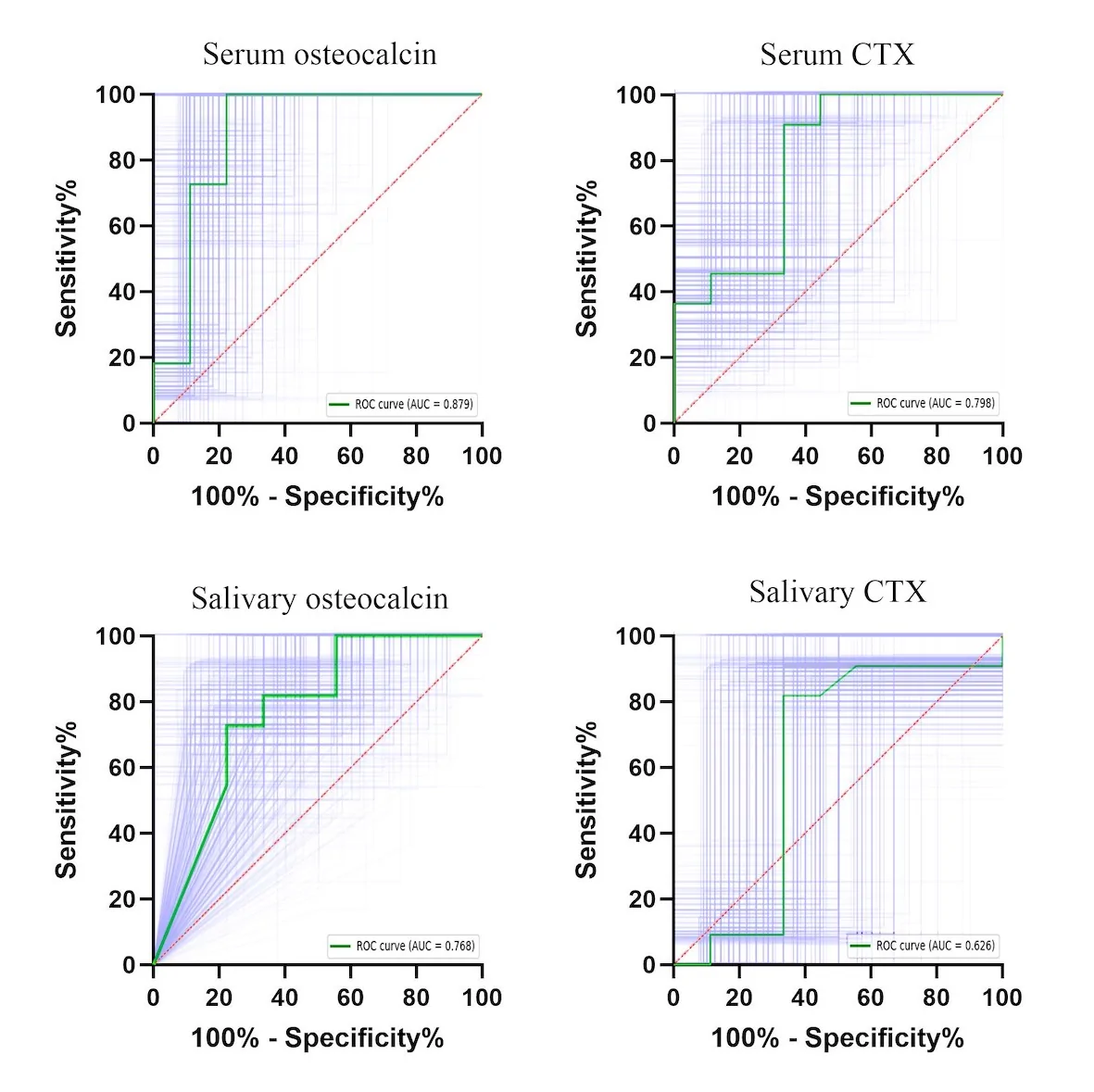
Receiver operating characteristic (ROC) curves for serum and saliva CTX and OC in distinguishing between MRONJ at-risk and MRONJ clinically present with bootstrapping.

## Data Availability

The data that support the findings of this study are available from the corresponding author upon reasonable request.
